# Transcriptional responses in the adaptation to ischaemia-reperfusion injury: a study of the effect of ischaemic preconditioning in total knee arthroplasty patients

**DOI:** 10.1186/1479-5876-8-46

**Published:** 2010-05-10

**Authors:** Terence Murphy, Pauline M Walsh, Peter P Doran, Kevin J Mulhall

**Affiliations:** 1 UCD Clinical Research Centre, UCD School of Medicine and Medical Sciences, Mater University Hospital, Dublin, Ireland; 2 Cappagh National Orthopaedic Hospital, Dublin, Ireland

## Abstract

**Background:**

Ischaemic preconditioning (IPC) has emerged as a method of reducing ischaemia-reperfusion injury. However, the complex mechanism through which IPC elicits this protection is not fully understood. The aim of this study was to investigate the genomic response induced by IPC in muscle biopsies taken from the operative leg of total knee arthroplasty patients in order to gain insight into the IPC mechanism.

**Methods:**

Twenty patients, undergoing primary total knee arthroplasty, were randomly assigned to IPC (n = 10) and control (n = 10) groups. Patients in the IPC group received ischaemic preconditioning immediately prior to surgery. IPC was induced by three five-minute cycles of tourniquet insufflation interrupted by five-minute cycles of reperfusion. A muscle biopsy was taken from the operative knee of control and IPC-treated patients at the onset of surgery and, again, at one hour into surgery. The gene expression profile of muscle biopsies was determined using the Affymetrix Human U113 2.0 microarray system and validated using real-time polymerase chain reaction (RT-PCR). Measurements of C-reactive protein (CRP), erythrocyte sedimentation (ESR), white cell count (WCC), cytokines and haemoglobin were also made pre- and post-operatively.

**Results:**

Microarray analysis revealed a significant increase in the expression of important oxidative stress defence genes, immediate early response genes and mitochondrial genes. Upregulation of pro-survival genes was also observed and correlated with a downregulation of pro-apoptotic gene expression. CRP, ESR, WCC, cytokine and haemoglobin levels were not significantly different between control and IPC patients.

**Conclusions:**

The findings of this study suggest that IPC of the lower limb in total knee arthroplasty patients induces a protective genomic response, which results in increased expression of immediate early response genes, oxidative stress defence genes and pro-survival genes. These findings indicate that ischaemic preconditioning may be of potential benefit in knee arthroplasty and other musculoskeletal conditions.

## Background

Ischaemic preconditioning has emerged as an extremely powerful method of protecting tissue against ischaemia-reperfusion injury [1]. It is an innate protective mechanism that increases a tissue's tolerance to prolonged ischaemia when it is first subjected to short bursts of ischaemia and reperfusion. It is thought to provide this protection by increasing the tissue's tolerance to ischaemia, thereby reducing oxidative stress, inflammation and apoptosis in the preconditioned tissue. The protective effects of ischaemic preconditioning have been demonstrated in animal models [2,3] and are now being investigated in human trials [4-8].

The complex mechanism through which IPC provides protection has only been partially elucidated. Studies have shown that IPC triggers the release of signalling molecules such as adenosine [3], bradykinin [9] and reactive oxygen species (ROS) [10]. The release of these molecules then activates protective signalling pathways involving kinases such as protein kinase C [11], PI-3K [12], tyrosine kinase [13] and MAPK kinases. This culminates in protection through reduced energy consumption, reduced oxidative stress, upregulation of heat shock proteins and inhibition of apoptosis with a resultant reduction in tissue injury.

Relatively little data describing the genomic response to ischaemic preconditioning in humans has been reported. Accordingly, we sought to investigate the effect of IPC in patients undergoing total knee arthroplasty. The primary objective of this study was to investigate the genomic response induced by IPC in muscle biopsies taken from the operative leg of total knee arthroplasty patients using microarray analysis. A secondary objective was to evaluate the effects of IPC on the systemic inflammatory response.

## Methods

### Study design and patient selection

Ethical approval for this study was granted by the ethics committee of the Cappagh National Orthopaedic Hospital, Dublin, Ireland. Informed consent was obtained from each patient before enrolment in the study. Patients undergoing primary knee arthroplasty (n = 20) were randomised to IPC (n = 10) and control (n = 10) groups, and patients were unaware of whether they were in the control or study group. Excluded from the study were (1) patients with abnormal ankle brachial indexes indicating poor vascular supply to the limb (2) patients with inflammatory arthropathies and (3) diabetic patients as there has been some correlation between oral sulphonurea therapy and preconditioning [14]. One patient was diagnosed with rheumatoid arthritis following recruitment and, therefore, was excluded from the study.

### Preconditioning protocol

**Figure 1:**
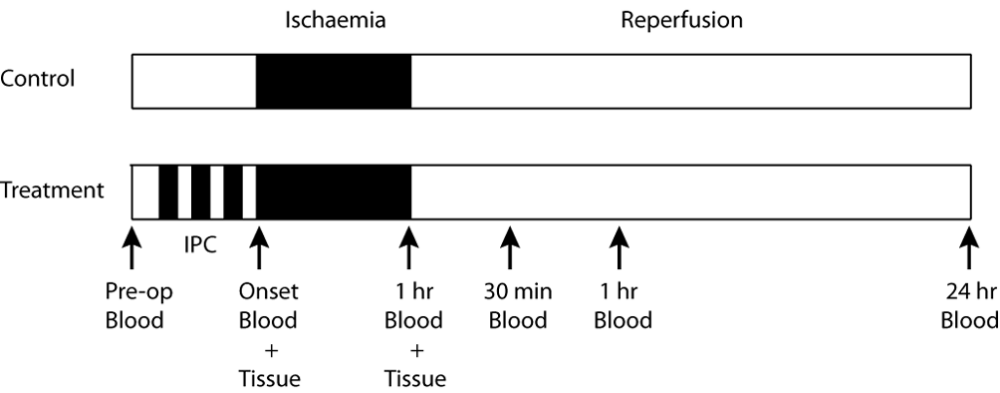
An overview of the study timeline and experimental approach.

All patients had a tourniquet placed on the upper thigh of the operative limb after the administration of spinal anaesthesia as per normal protocol for knee arthroplasty surgery in our unit. The ischaemic preconditioning stimulus consisted of three five-minute periods of tourniquet insufflation on the upper thigh of the operative limb, interrupted by five minute periods of reperfusion. The pressure to which the tourniquet was insufflated for preconditioning was determined in relation to systolic blood pressure for each patient. The tourniquet was set 100 mm Hg above the patient's systolic BP to ensure ischaemic conditions. The control group simply had tourniquet insufflation as normal at the start of surgery. This preconditioning protocol has been used previously in human trials involving the upper and lower limb [15,16]. An overview of the experimental approach is provided in Figure 1.

### Blood sampling and serological analysis

Pre-operative blood samples were collected as per routine protocol. Peripheral blood samples were obtained from the antecubital fossa of the upper limb at the initiation of surgery and at 1 hour of ischaemia to coincide with the muscle sampling. Blood was then obtained 30 min, 1 hour and 24 hours following tourniquet release to investigate the effect of reperfusion (Figure 1). Blood samples were centrifuged at 2000 × g for 15 min and the resulting serum samples were stored at -80°C. Serum samples were analysed for cytokine expression using the MSD Human Pro-Inflammatory 9-Plex Ultra-Sensitive Kit (Meso Scale Discovery, USA), according to the manufacturer's instructions. Blood samples were also analysed for haemoglobin, ESR, CRP and white cell count.

### Muscle sampling and RNA extraction

Intra-operative sampling was used to obtain muscle biopsies from the quadriceps muscle. Muscle biopsies were taken from the operative knee at the immediate onset of surgery (t = 0), and again, at one hour into the surgery (t = 1). Biopsies were rapidly frozen in liquid nitrogen and stored at -80°C. For extraction of total RNA, muscle biopsies (~100 mg) were added directly to a ceramic mortar containing liquid nitrogen and ground to a fine powder using a pestle. An aliquot of ice-cold TRI reagent (Sigma, Ireland) was added to the ground muscle powder, mixed using a vortex, and immediately homogenised on ice using a Polytron homogeniser (Kinematica, USA). Total RNA was isolated from the homogenised solution according to the manufacturer's instructions (Sigma, Ireland). RNA integrity was assessed using an Agilent 2100 Bioanalyser (Agilent Technologies, Germany).

### Affymetrix GeneChip hybridization, scanning and data analysis

The gene expression profile of muscle biopsies taken from four control and four IPC-treated patients was determined using the Affymetrix Human U113 2.0 microarray system (Affymetrix, Santa Clara, CA). Sample amplification, labelling, hybridisation and detection were carried out by Almac Diagnostics, Craigavon, N. Ireland. Briefly, 2 μg total RNA was reversed transcribed to cDNA, subjected to amplification and labelling followed by hybridisation to an array for 16-18 hours at 45°C. The array was then washed and stained with streptavidin-phycoerythrin on the GeneChip^® ^Fluidics Station 450, and scanned using the GeneChip^® ^Scanner 3000. The Rosetta Error Model was applied to the raw data to generate intensity values. Gene filtering was then applied to identify significantly differentially regulated genes. Filters included: intensity p-value filter, background filter, fold change filter and signature p-value filter. Gene lists were analysed using DAVID 2.0 [17] and Ingenuity Pathway Analysis (Ingenuity^® ^Systems, http://www.ingenuity.com). The results of microarray analysis were validated by real-time PCR. The following 5 genes were validated by real-time PCR: early growth response 1 (*egr1*), cellular oncogene c-fos (*fos*), jun oncogene (*jun*), pyruvate dehydrogenase kinase 4 (*pdk4*) and heat shock 22 kDa protein 8 (*hspb8*). The nucleotide sequences of the primers used for real-time PCR are given in Table 1.

**Table 1:** Forward and reverse primers used for real-time PCR validation of microarray results

Gene	Primer Sequence
EGR1	F: 5'-AGCCCTACGAGCACCTGAC-3'
	R: 5'-AGCGGCCAGTATAGGTGATG-3'
PDK4	F: 5'-GTCCCTACAATGGCACAAGG-3'
	R: 5'-GGTTCATCAGCATCCGAGTAG-3'
JUN	F: 5'-GAGCGGACCTTATGGCTACA-3'
	R: 5'-TGAGGAGGTCCGAGTTCTTG-3'
FOS	F: 5'-CAAGCGGAGACAGACCAAC-3'
	R: 5'-GAGCTGCCAGGATGAACTC-3'
HSPB8	F: 5'-AGCCAGAGGAGTTGATGGTG-3'
	R: 5'-TGCAGGAAGCTGGATTTTCT-3'
GAPDH	F: 5'-GAGTCAACGGATTTGGTCGT-3'
	R: 5'-TTGATTTTGGAGGGATCTCG-3'

* F, forward; R, reverse.

### Complementary DNA synthesis and real-time PCR

Genomic DNA was removed from RNA samples using a DNA-*free*™ kit (Applied Biosystems, UK). RNA was then converted to complementary DNA (cDNA) using Enhanced Avian Reverse Transcriptase (Sigma). cDNA then served as template for Real-Time PCR, which was conducted using QIAGEN QuantiTect SYBR Green PCR kit. Gene expression was measured using absolute quantification, normalised to control and glyceraldehyde 3-phosphate dehydrogenase (*gapdh*) expression resulting in mean fold change.

### Statistical Analysis

Data are given as a mean +/- standard deviation. Real-time PCR data were analysed by an unpaired t-test to determine a significant difference between sample means. Serological data were analysed by a one-sample t-test and a paired two-sample t-test. Differences were considered significant if *P *< 0.05.

## Results

To uncover the genomic response induced by ischaemic preconditioning, we analysed global gene expression levels in muscle biopsies taken from total knee arthroplasty patients using the Affymetrix Human U113 2.0 microarray system. Using RNA isolated from muscle biopsies taken from the operative leg at the immediate onset of surgery (t = 0), and again, at one hour into the surgery (t = 1), the gene expression profiles of control and preconditioned patients were compared. The analysis of gene expression patterns at the onset of surgery allowed for the identification of changes resulting from the preconditioning stimulus, which was performed immediately prior to surgery. The analysis of gene expression patterns at one hour into surgery permitted the identification of protective signalling, induced by IPC, which occurred at 1 hour into surgery.

All patients had an uneventful surgery and there were no adverse complications noted in the immediate post-operative period. There was no significant difference found between the two groups regarding patient demographics (Table 2). All patients underwent primary elective knee arthroplasty. None of the patients had any severe deformity or complicating clinical scenarios which required prolonged procedures to obtain good surgical outcome. The duration of tourniquet application time in all patients ranged from 68 to 87 minutes.

**Table 2:** Patient demographics

	Control (n = 9)	IPC (n = 10)	*P*
Age (years)	70.8 (+/- 7.3)	66.4 (+/- 9.6)	0.28
Sex ratio (M:F)	2:7	6:4	0.61

### Differential gene expression at the onset of surgery (t = 0)

**Figure 2:**
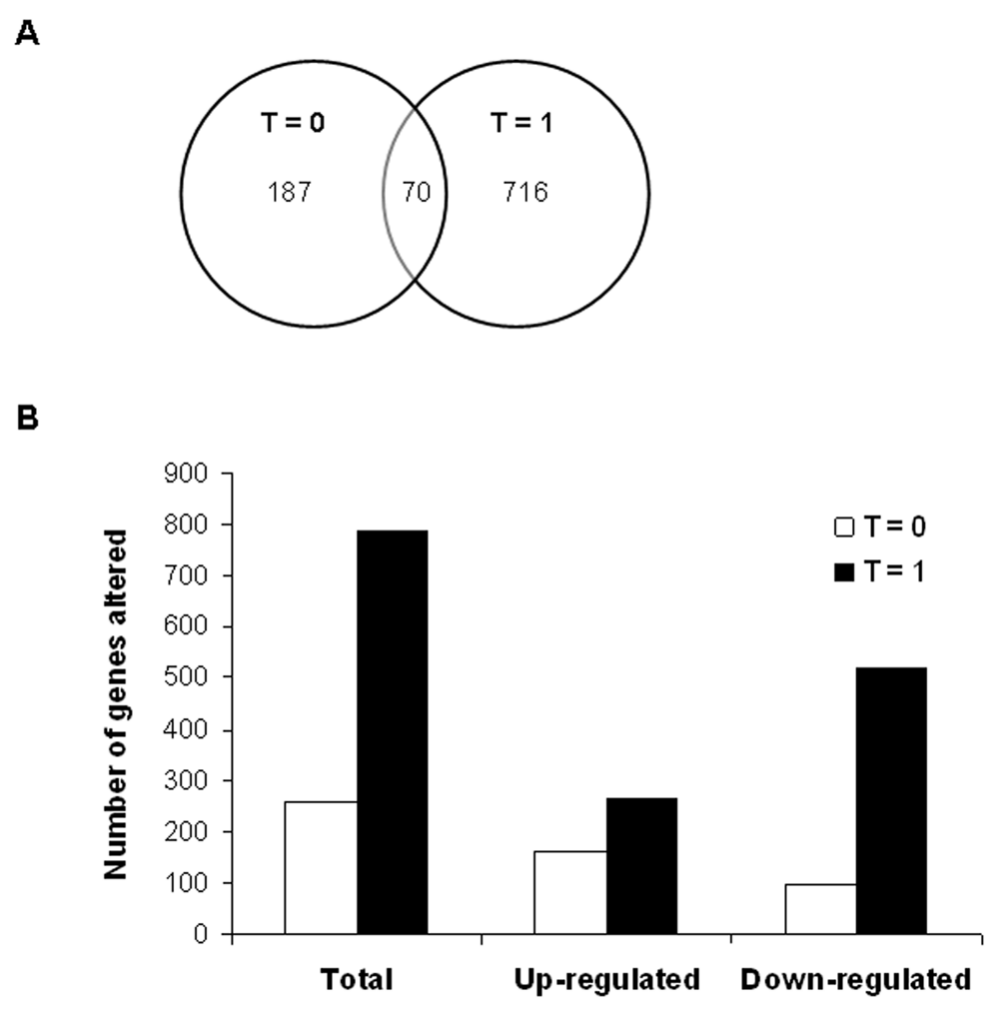
**Analysis of microarray data**. (A) Venn diagram depicting the overlap of differentially expressed genes at the onset of surgery (t = 0) and at 1 hour into surgery (t = 1). (B) Numbers of genes demonstrating a minimum of 1.5 fold-change in expression at the two time-points studied.

**Figure 3:**
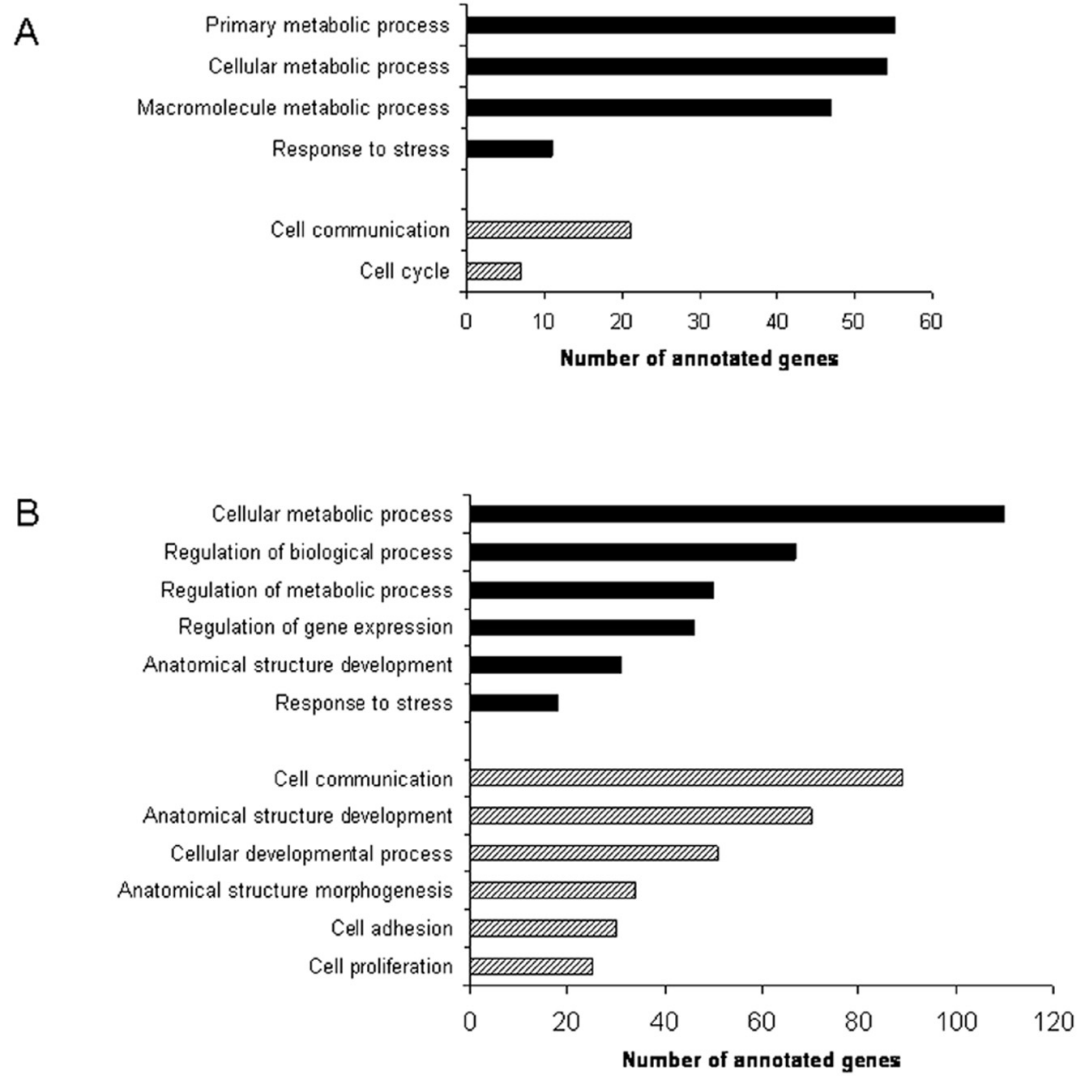
**Annotation of microarray data using Gene Ontology**. A bar chart representing the numbers of genes differentially expressed at the immediate onset of surgery (A) and at 1 hour into surgery (B) classified according to biological process. Genes that were found to be upregulated are shown in black and genes found to be downregulated are shown in white.

Firstly, the changes in gene expression which occurred at the onset of surgery (t = 0) were analysed. This analysis revealed that 257 genes were significantly differentially regulated >1.5 fold in the IPC group as compared to the control group. Of these 257 genes, 162 genes were up-regulated >1.5 fold while 95 genes were downregulated >1.5 fold (Figure 2). Gene ontology (GO) analysis was performed to gain a comprehensive understanding of the gene classes that were differentially regulated in the IPC group. Genes were analyzed by their GO annotations, including biological process, molecular function and cellular component categories. Ontology analysis, preformed using DAVID 2.0 revealed an upregulation of genes relating to metabolic processes, mitochondrial biogenesis/organisation and response to stress at this timepoint (Figure 3 and Table 3).

**Table 3:** Genes up-regulated in IPC patients compared to control patients at the onset of surgery (t = 0)

Gene name	Symbol	Public ID	Fold change	*P*
**Mitochondrial**				
COX18 cytochrome c oxidase assembly homolog	COX18	AI769476	1.71	0.036
COX11 cytochrome c oxidase assembly homolog	COX11	AI376724	1.87	0.008
Uncoupling protein 3	UCP3	NM_003356	1.99	0.037
Translocase of inner mitochondrial membrane 10	TIMM10	AF152354	1.54	0.001
Mitochondrial ribosomal protein L43	MRPL43	N74662	1.90	0.007
Pyruvate dehydrogenase kinase 4	PDK4	AL832708	3.57	0.022
**Other**				
BCL2/adenovirus E1B 19 kDa interacting protein1	BNIP1	NM_013979	1.55	0.001

* A positive number indicates elevated expression (fold change) in skeletal muscle tissue of IPC-treated patient compared to skeletal muscle tissue of control patient.

### Differential gene expression at 1 hour into surgery (t = 1)

We next analysed those genes that were differentially regulated at 1 hour into surgery (t = 1). These data revealed a significantly higher number of differentially regulated genes compared to the onset of surgery (Figure 2). A total of 786 genes were differentially expressed >1.5 fold at this time point, 519 genes were downregulated while 267 genes were up-regulated (Figure 2). Ontology analysis revealed a downregulation in genes related to cell communication, developmental processes, cell adhesion and cell proliferation (Figure 3). An upregulation in the expression of genes related to the regulation of metabolic processes, biological processes and gene expression was observed (Figure 3). Increased expression of genes involved in the response to stress including oxidative stress, and the regulation of cell death was also observed (Table 4).

**Table 4:** Genes up- or down-regulated in IPC patients compared to control patients at 1 hour into surgery (t = 1)

Gene name	Gene symbol	Public ID	Fold change	*P*
**Immediate early genes**				
Early growth response 1	EGR1	AV733950	2.84	0.001
Myc proto oncogene protein	MYC	NM_002467	2.58	0.038
Cellular oncogene c-fos	FOS	BC004490	2.11	0.018
Immediate early response 2	IER2	NM_004907	1.58	0.034
Jun oncogene	JUN	BC002646	1.43	0.001
				
**Oxidative stress defence**				
Catalase	CAT	AU147084	2.14	0.017
Glutathione S-transferase theta 1	GSTT1	AL359937	2.69	0.025
Sequestosome 1	SQSTM1	AW293441	2.04	0.018
				
**Chaperone/Survival**				
DnaJ (Hsp40) homolog, subfamily B, member 6	DNAJB6	AF080569	1.45	0.042
Heat shock 22 kDa protein 8	HSPB8	BF109740	1.83	0.001
BCL2/adenovirus E1 B 19 kDa interacting protein 1	BNIP1	NM_013979	1.50	0.004
BCL6 co-repressor	BCOR	AF317391	1.74	0.047
				
**Anti-apoptotic**				
Caspase 8	CASP8	BF439983	-2.00	0.045
Caspase 7	CASP7	NM_001227	-1.31	0.026
				
**Mitochondrial**				
Uncoupling protein 3	UCP3	NM_003356	2.41	0.001
Pyruvate dehydrogenase kinase 4	PDK4	AL832708	2.99	0.009

* A positive number indicates elevated expression (fold change) in skeletal muscle tissue of IPC-treated patient compared to skeletal muscle tissue of control patient. A negative number indicates decreased expression (fold change) in skeletal muscle tissue of IPC-treated patient compared to skeletal muscle tissue of control patient.

### Validation of microarray analysis using real-time PCR

**Figure 4:**
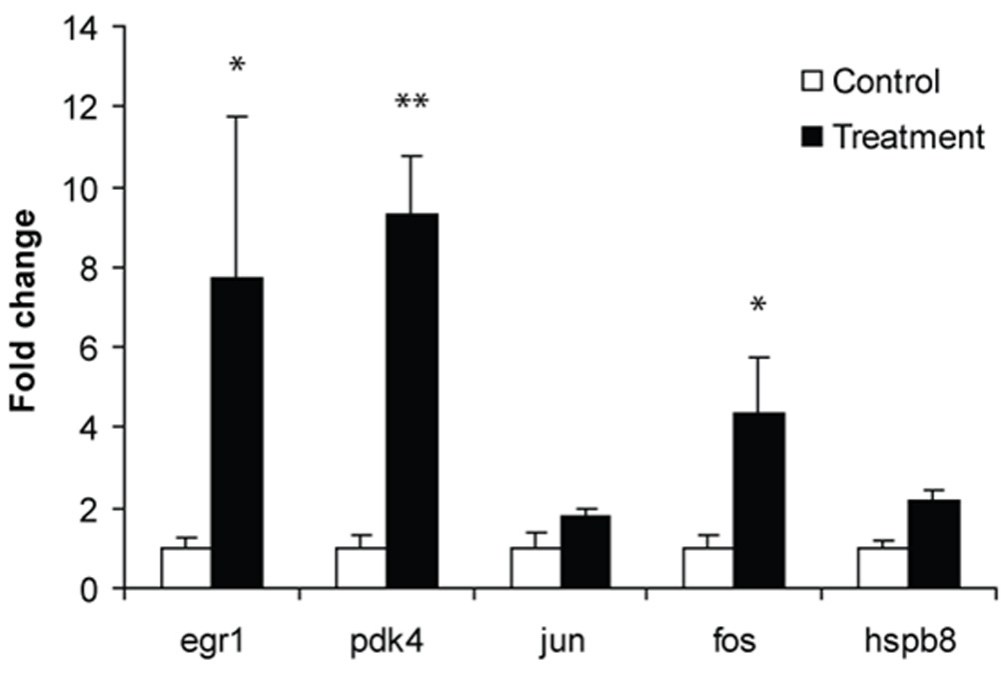
**Validation of microarray data using RT-PCR**. Gene expression patterns of five selected genes in skeletal muscle biopsies of control and preconditioned patients as determined by RT-PCR. Values are the mean fold difference from control. * = P < 0.05; ** = P < 0.01 for control group versus IPC group.

The results of microarray analysis were validated by real-time PCR in an additional 4 patients. Real-time PCR was performed on five selected genes (*egr1*, *fos*, *pdk4*, *jun*, and *hspb8*), a number of which have previously been associated with the ischaemic preconditioning mechanism, i.e. *egr1*, *fos *and *jun*. Expression analysis of the five chosen genes by real-time PCR correlated with our array data, and showed that the expression levels of *egr1*, *fos *and *pdk4 *in control and preconditioned samples were significantly different when analysed by both methods (Figure 4).

### Systemic effects of IPC

**Figure 5:**
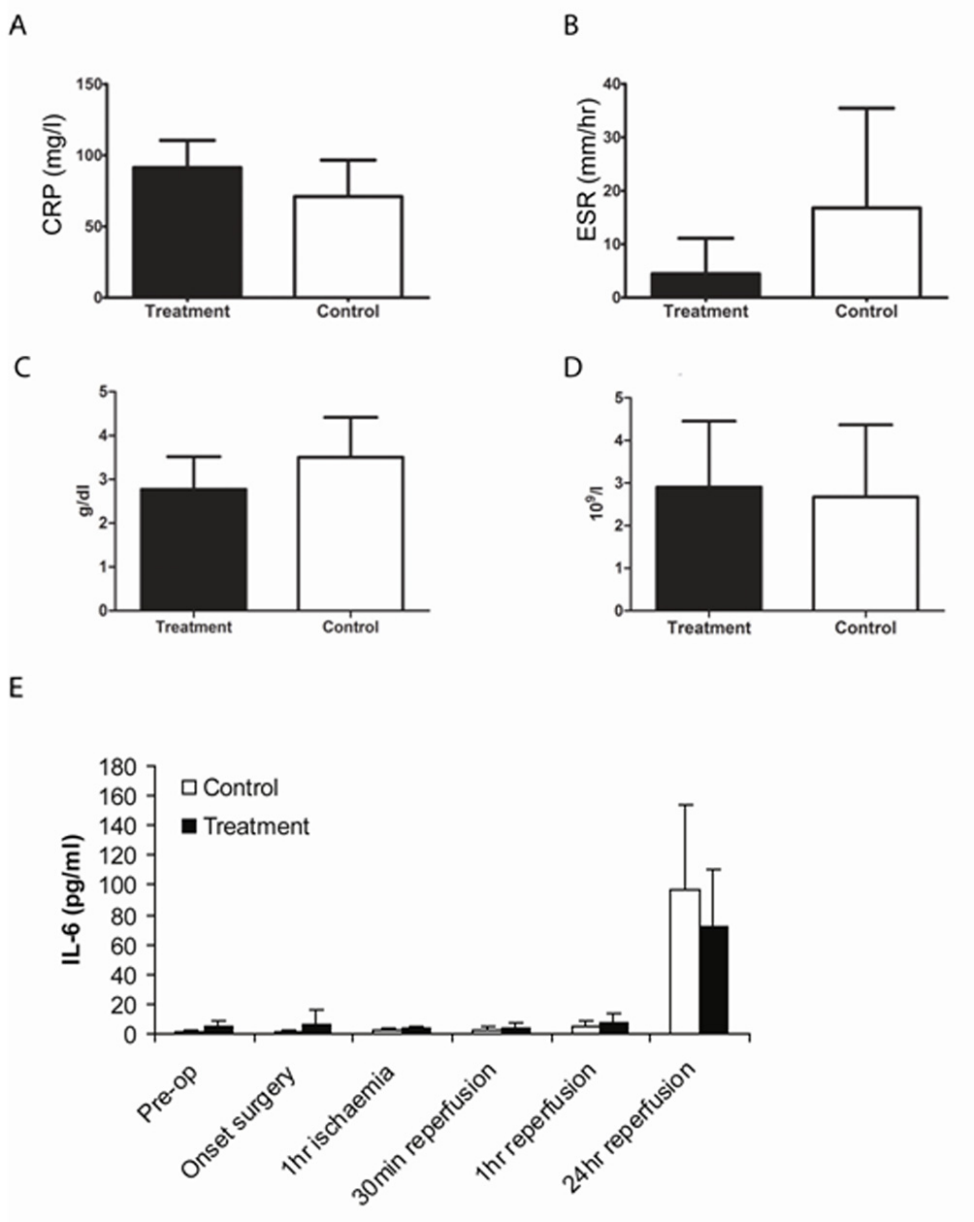
**Analysis of serological data**. Changes in the level of CRP (A), ESR (B), haemoglobin (C) and WCC (D) in control and ischaemic preconditioned patients at 24 hours post-surgery. Pre-, intra- and post-operative levels of IL-6 in control and preconditioned patients (E). Data are represented as means +/- the standard deviation.

No statistically significant difference was found between the control and treatment groups with regard to circulating levels of CRP, ESR and white blood cell count (Figure 5A, B, C). There was a reduction in haemoglobin loss in the treatment group at 24 hours post-reperfusion but this reduction was not statistically significant (p < 0.081; Figure 5D). Mean levels of the pro-inflammatory cytokines IL-8, TNF-alpha, INF-gamma, IL-1-beta, IL-2, IL-10, IL-12p70, GM-CSF were also measured and again no statistically significant differences were demonstrated. IL-6 levels were significantly increased at 30 min (1.35 pg/ml ± 1.7, p < 0.037) and 1 hour (3.11 pg/ml ± 3.25, p < 0.014) post-reperfusion in the control group, and at 24 hours post-reperfusion in both groups (control 95.1 pg/ml ± 56.4, 95%, p < 0.0005; treatment 67.5 pg/ml ± 37.8, p < 0.0005). The mean IL-6 level in the control group at 24 hours of reperfusion was higher than that of the IPC group (95.1 pg/ml ± 56.4 v 67.5 pg/ml ± 37.8), however, this difference was not statistically significant (Figure 5E).

## Discussion

Ischaemic preconditioning has been shown to protect against ischaemia-reperfusion injury in both animal models and human studies [1,3,15,18], however, the signalling mechanisms responsible remain unclear. To date, relatively little data describing the genomic response to ischaemic preconditioning in humans has been reported. Therefore, to identify the genomic response induced by ischaemic preconditioning, we analysed gene expression patterns in a cohort of total knee arthroplasty patients using the Affymetrix Human U113 2.0 microarray system.

While such a cohort of patients is unlikely to develop serious complications of ischaemia-reperfusion, this study provided a model for investigating the local and systemic effects of ischaemic preconditioning, as standard practice for total knee arthroplasty in our institution already involves the application of a tourniquet for the duration of the operation. In this study, ischaemic preconditioning was induced by three five-minute cycles of tourniquet insufflation on the operative lower limb interrupted by five-minute cycles of reperfusion; this preconditioning protocol has previously been shown to be effective in other clinical studies [15,16].

We investigated the mechanism of local IPC by comparing the gene expression profile of muscle biopsies taken from the operative leg of control and IPC-treated patients using microarray analysis. IPC was found to induce a gene expression profile which was indicative of a protective genomic response in muscle biopsies taken from IPC-treated patients. A comparison of the gene expression profiles of the control and IPC groups indicated that the effect of ischaemic preconditioning was correlated with increased expression of genes involved in immediate early response, defence against oxidative stress, pro-survival functions, and a decrease in gene expression associated with cell death.

### IPC triggers the expression of early response genes

In the present study, increased expression of immediate early response genes was shown to be associated with the protective response induced by IPC. This was exemplified by an upregulation in the expression of *egr1*, *ier2*, *c-fos*, *c-jun *and *myc*. Immediate early response genes are a group of genes that are activated transiently and rapidly in response to a wide variety of cellular stimuli. Furthermore, a number of these genes have previously been reported to be involved in the adaptation to ischaemia and in the IPC mechanism [19,20]. In a rat model of IPC, increased expression of *c-fos *and *myc *was found to be associated with cardioprotection as evidenced by improved ventricular function and reduced infarct size [19]. More recently, increased expression of *egr1 *was associated with a predicted cardioprotective phenotype induced by intraoperative ischaemia-reperfusion [21]. The high incidence of early response gene expression indicates that the induction of these genes may be an important element of the protective response induced by IPC.

### IPC induces stress response and prosurvival gene expression

The cytoprotective abilities of anti-oxidant proteins induced by IPC are well documented in *in vitro *and animal models [19,22,23]. In the present study, microarray analysis revealed increased expression of anti-oxidant genes in IPC-treated patients following one hour of ischaemia, including catalase and glutathione S-transferase theta 1. Increased ROS generation occurs in ischaemic tissue upon reperfusion. An important element of the cellular defence against ROS is the induction of antioxidant enzymes and detoxifying enzymes such as catalase and glutathione S-transferase. Catalase functions in the decomposition of hydrogen peroxide to water and oxygen while glutathione S-transferases catalyze the conjugation of reduced glutathione to a variety of electrophilic and hydrophobic compounds. Nuclear factor-erythroid 2-related factor 2 (Nrf2) is a transcription factor and an important regulator of the cells response to oxidative stress [24]. It regulates the expression of a network of cytoprotective enzymes and has recently been shown to be involved in the ischaemic preconditioning mechanism [25,26]. Pathway analysis revealed induction of a number genes involved in Nrf2 signalling in IPC-treated patients, including catalase, glutathione S-transferase, sequestosome 1, jun and fos. Nrft2 signalling has recently been shown to protect against ischaemia-reperfusion injury in both a kidney cell line and in liver biopsies [25,26]. Results of our study give further support to the idea that Nrf2 signalling is an important protective signalling pathway activated by IPC.

Analysis of microarray data demonstrated increased expression of genes with pro-survival or chaperone functions in IPC patients. Increased expression of heat shock protein 22 kDa protein 8, BCL2/adenovirus E1B 19 kDa interacting protein 1, and BCL6 co-repressor and DnaJ (Hsp40) homolog, subfamily B, member 6 was observed in IPC-treated patients. Studies have shown that heat shock proteins play a key role in the protection provided by IPC, in particular HSP70 and HSP27 [27-29]. The induction of pro-survival gene expression was also associated with a reduction in pro-apoptotic gene expression (caspase 7 and 8) suggesting that IPC may modulate both cell survival and cell death pathways.

### The systemic effect of IPC

Ischaemic preconditioning, induced by transient ischaemia of a limb, has been shown to protect remote organs against the effects of ischaemia-reperfusion injury [15,18,30]. In a study of children undergoing cardiopulmonary bypass surgery, patients that received remote IPC (via transient ischaemia of the leg) had less cardiac and pulmonary insult [18]. Similarly, in adult patients, decreased serum troponin levels were detected after cardiopulmonary bypass surgery in those patients that received remote IPC via transient ischaemia of the upper arm [15]. It has also been proposed that remote preconditioning may protect against ischaemia-reperfusion injury through a potent suppression of inflammatory signals. Evidence to support this has been demonstrated in healthy volunteers where ischaemic preconditioning of the upper arm has been shown to provide remote protection in the form of reduced inflammatory cell activation and reduced endothelial dysfunction in the contralateral arm [31], and to suppress pro-inflammatory gene expression in circulating leukocytes [32].

In this study, we investigated the effect of ischaemic preconditioning on the systemic inflammatory response to ischaemia-reperfusion in our cohort of total knee arthroplasty patients (n = 20). While the patients in this cohort were unlikely to suffer serious complications of ischaemia-reperfusion, a statistically significant increase in the circulating levels of IL-6 was observed in both groups at 24 hours post-reperfusion indicating a post-operative systemic inflammatory response occurred in both patient groups. While skeletal muscle is relatively resistant to ischaemic-reperfusion injury, studies have shown that tourniquet-induced ischaemia-reperfusion leads to systemic activation of PMNs and T cells [16,30]. In the present study, no significant difference in the mean levels of circulating cytokines was observed between patient groups. However, IPC patients had a tendency for a reduction in IL-6 and ESR at 24 hours post-reperfusion indicating that IPC may attenuate the post-operative inflammatory response in these patients. Other studies have shown that a local IPC stimulus, induced via transient ischaemia of the lower limb, can modulate the systemic inflammatory response following ischaemic-reperfusion in a rat model of limb ischaemic-reperfusion and in patients undergoing cruciate ligament reconstruction [16,30]. While these studies, and the current study, have shown that local IPC exerts distant anti-inflammatory effects, it is important to note that local and remote IPC are two separate forms of preconditioning and that the signalling mechanisms underlying both forms are not entirely similar.

## Conclusions

In summary, the findings of this study show that IPC induces a protective genomic response in total knee arthroplasty patients. The protective effect of IPC was associated with increased expression of genes involved in immediate early response, defence against oxidative stress and pro-survival functions. This study also served as a pilot study to demonstrate the safety of this technique in TKA patients. Results of this study indicate that IPC may be of potential benefit in this and other musculoskeletal conditions.

## Competing interests

The authors declare that they have no competing interests.

## Authors' contributions

TM, PPD and KJM conceived and designed the experiments. TM performed the preconditioning protocol and collected patient samples. TM and PMW carried out the experimental work including the extraction of RNA, validation of microarray data by real-time PCR and the analysis of serum samples. The microarray experiment and the analysis of array data were carried out by Almac Diagnostics, Craigavon, N. Ireland. PMW was responsible for the annotation of the microarray data and the preparation of the manuscript. All authors read and approved the final manuscript.
